# Expanding Access to Presurgical Cleft Care: Digital Nasoalveolar Molding with Clear Aligners in a Rural Low-Income Population †

**DOI:** 10.3390/children12091231

**Published:** 2025-09-15

**Authors:** Diogo C. Frazao, Miguel A. C. Salgado, Ryan J. Cody, Elizabeth M. Kay, Henrique Pretti, G. Dave Singh, Luiz A. Pimenta

**Affiliations:** 1 Department of Science and Technology Applied to Dentistry, Institute of Science/Technology, Universidade Estadual Paulista “Júlio de Mesquita Filho” (Unesp), São José dos Campos, São Paulo 12245-000, Brazil; frazao@unesp.br; 2 Department of Biosciences and Oral Diagnosis, Institute of Science and Technology, Sao Paulo State University, São Paulo 05508-020, Brazil; miguel.angel@unesp.br; 3 Department of Orthodontics, School of Dentistry, University of Texas, Houston, TX 78712, USA; rxcody@texaschildrens.org; 4 Chapel Hill Adams School of Dentistry, University of North Carolina, Chapel Hill, NC 27599, USA; ekay6370@gmail.com; 5 Department of Orthodontics, Federal University of Minas Gerais, Belo Horizonte 31270-901, Brazil; bhpretti@gmail.com; 6 Sleep Medicine, Stanford University, Stanford, CA 94305, USA; drsingh@davesingh.com; 7 Division of Prosthodontics, College of Dental Medicine Columbia University, New York, NY 10032, USA

**Keywords:** cleft lip and palate, digital nasoalveolar molding, pediatric craniofacial orthopedics

## Abstract

**Highlights:**

This study reports the application of a digitally guided presurgical molding protocol using clear aligners in infants with unilateral cleft lip and palate. The method proved feasible and safe, producing significant short-term reductions in cleft width with only mild, transient skin reactions observed. While qualitative improvements in nasal symmetry were observed, objective measures and long-term outcomes have yet to be determined. By leveraging digital planning and simplified appliance delivery, this approach has the potential to broaden access to early cleft care, though scalability and cost-effectiveness require further evaluation.

**What are the main findings?**

- Digital NAM using clear aligners significantly reduced anterior (–5.38 mm) and posterior (–3.39 mm) cleft widths.
- Intermolar width increased (+1.23 mm) and intercanine width remained stable, indicating preserved maxillary arch form during treatment.

**What is the implication of the main finding?**

- A digitally planned, low-contact NAM clear aligner protocol can achieve effective presurgical molding with minimal in-office visits.
- This model supports broader access to cleft care in underserved and remote populations by enabling caregiver participation and digital workflow scalability.

**Abstract:**

**Background:** Presurgical nasoalveolar molding (NAM) improves outcomes in infants with cleft lip and palate by guiding alveolar segment alignment and enhancing nasal symmetry prior to primary lip repair. However, traditional NAM protocols require frequent clinical visits and specialized expertise, limiting access for families in rural and low-resource settings. **Objective**: This retrospective clinical study evaluated the feasibility and clinical outcomes of a digitally guided NAM approach using thermoformed clear aligners in infants with unilateral complete cleft lip and palate. **Material and Methods:** Twenty-five neonates residing in rural regions were treated over a 20-week pre-surgical period using a digital workflow that included intraoral scanning, 3D model design, and sequential aligner fabrication. The protocol minimized the number of in-office visits while engaging caregivers in home-based appliance management. Anatomical changes were assessed using 3D models at baseline and at treatment completion. **Results:** Significant reductions were observed in anterior cleft width (mean decrease: 5.38 mm, 95% CI: –7.58 to –3.18, *p* < 0.001) and posterior cleft width (mean decrease: 3.39 mm, 95% CI: –4.79 to –1.99, *p* < 0.001). Intermolar distance increased by 1.23 mm (*p* = 0.036), while intercanine width remained stable (*p* = 0.515), indicating preservation of maxillary arch form. Surgeons reported improved nasal symmetry and tissue alignment at the time of lip repair. **Conclusions:** This digitally planned NAM clear aligner protocol demonstrated clinical feasibility and effectiveness in reducing cleft width during the pre-surgical period. Findings should be interpreted with caution, given the retrospective design, lack of a control group, and absence of objective nasal outcome measures. Further studies are recommended to assess long-term outcomes and broader implementation potential.

## 1. Introduction

Cleft lip and palate represent some of the most prevalent congenital craniofacial anomalies globally, affecting roughly 1 in every 700 live births [1]. The severity of these defects can vary considerably, often requiring individualized, multidisciplinary management from infancy through adolescence. Among the earliest interventions available is nasoalveolar molding (NAM)—a presurgical orthopedic approach designed to approximate the maxillary segments, support nasal symmetry, and reduce cleft severity prior to primary lip repair [2,3,4,5,6].

NAM has been widely recognized for its ability to facilitate surgical correction by improving tissue alignment, reducing intraoperative tension, and potentially minimizing the need for secondary procedures [5,6]. Despite its clinical advantages, the widespread implementation of traditional NAM is hindered by several barriers, particularly in rural areas and low- and middle-income countries (LMICs). These challenges include the need for weekly in-person adjustments, extended travel distances for families, and a limited number of clinicians trained in NAM protocols [7,8,9].

To address these limitations, we developed a digitally guided NAM approach utilizing thermoformed clear aligners, combined with lip taping and a separate nasal protractor. In selecting the appliance design, we considered the specific needs of infants and their caregivers [10,11,12,13]. Clear aligners were chosen over other digital appliances because they are lightweight and generally more comfortable for the infant, can be fabricated with relative ease and precision using 3D printing workflows, and are simpler for caregivers to clean and maintain compared with bulkier conventional devices [10,14,15]. These practical advantages made clear aligners a suitable option for presurgical molding in this population. This protocol minimizes the frequency of clinic visits by empowering caregivers to manage appliance changes at home and leverages 3D design and printing technologies to standardize treatment delivery. It was specifically designed to improve access to presurgical cleft care in resource-constrained and geographically remote settings [7,9,16,17].

The purpose of this study was to evaluate the feasibility and early clinical outcomes of this digital clear aligner NAM technique in a rural, low-income population of infants with unilateral complete cleft lip and palate. We hypothesized that this approach could offer a scalable alternative to conventional NAM, capable of producing clinically meaningful anatomical improvements ahead of primary surgical intervention.

## 2. Materials and Methods

This retrospective clinical study was conducted through the Postgraduate Program in Science and Technology Applied to Dentistry at São Paulo State University (UNESP), in collaboration with the Department of Orthodontics at the Federal University of Minas Gerais (UFMG), Brazil. The clinical protocol was developed based on prior experience in craniofacial cleft care and digital treatment planning within interdisciplinary teams.

Ethical approval was obtained from the Committee of Ethics and Clinical Research at the Federal University of Minas Gerais (approval number: CAAE 1011619.1.0000.5149). From 2017 to 2019, twenty-five neonates diagnosed with unilateral complete cleft lip and palate were treated using a digitally guided nasoalveolar molding (NAM) protocol involving clear aligners. All patients resided in rural areas, with an average distance of over 100 km from the treatment center. Inclusion criteria included age under 30 days at the start of treatment and absence of syndromic conditions or craniosynostosis. No a priori sample size calculation was undertaken; the study population reflects all eligible cases treated during the study period. To aid interpretation, 95% confidence intervals are reported for all comparisons (Figure S1).

### 2.1. Clinical Protocol

#### Initial Evaluation and Impressions

**Figure 1:**
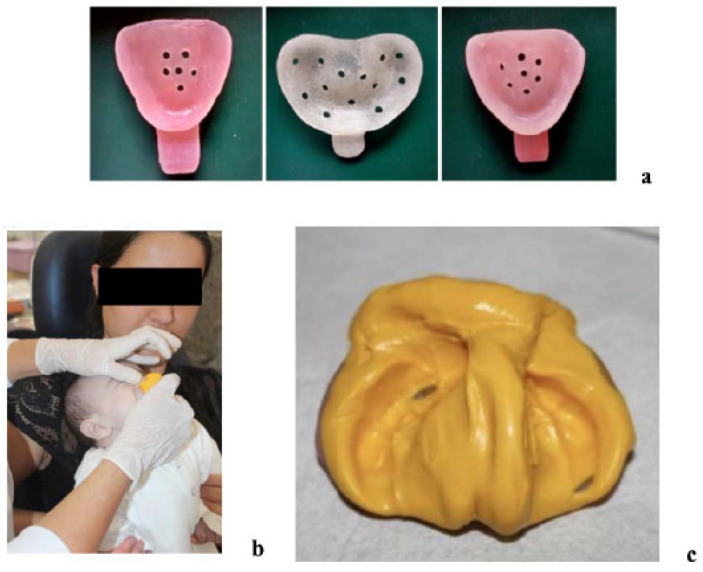
Impression procedures for NAM clear aligner therapy. (**a**) Prefabricated impression trays fabricated with Triad acrylic. (**b**) Intraoral impression taken with the mother securely holding the child. The moment reflects when the impression was removed. (**c**) Maxillary arch impression obtained using 3M Express STD Putty.

At the first clinical appointment, caregivers were educated on the treatment process, home responsibilities, and expected outcomes. Informed consent was obtained following a pre-treatment survey. Clinical documentation included extraoral and intraoral photographs, along with a maxillary impression using putty material (Express STD Putty; 3M) and a prefabricated tray (Triad; Dentsply Sirona). All impressions were performed with the infant held in a prone position, with the head slightly lower than the body, to reduce the risk of aspiration. The impression captured the alveolar ridge, vestibular sulcus, and palatal shelves (Figure 1).

### 2.2. Digital Scanning and Treatment Planning

**Figure 2:**
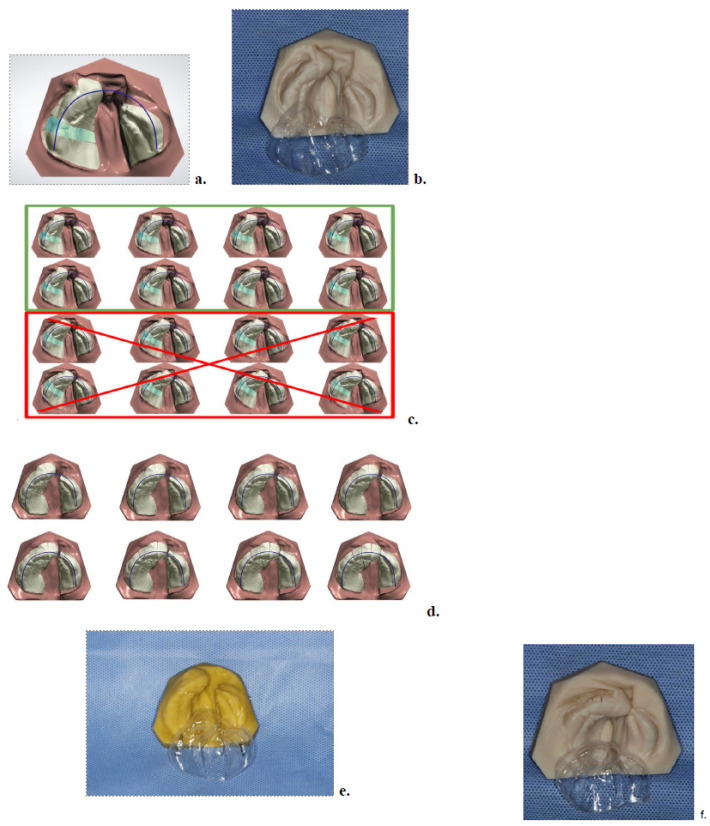
Digital treatment planning and clear aligner fabrication. (**a**) Initial digital model in OrthoPlanner, showing the desired arch form (outlined in purple) and segmentation of the greater maxillary segment into six pieces. (**b**) First NAM clear aligner. All aligners were fabricated from their corresponding resin models using a thermoplastic process. (**c**) Only the first half of the aligners (1–8) were printed and delivered; aligners 9–16 were not fabricated because the patient’s growth would outpace the volumetric dimensions of the initial impression. (**d**) Following completion of the first half of aligners, a new impression was obtained, serving as the basis for a Phase II digital treatment plan. (**e**) First aligner of Phase II treatment. (**f**) Final NAM clear aligner with its corresponding resin model, used as a retainer until the patient underwent surgery.

After disinfection, impressions were scanned using a desktop scanner (R700, TRIOS^®^; 3Shape, A/S, Copenhagen, Denmark), producing a high-resolution STL file. The scan was imported into OrthoAnalyzer software (3Shape, version 2019.1) to generate a digital model, which was then transferred to OrthoPlanner for treatment planning. The greater maxillary segment was digitally segmented into six parts to simulate future deciduous teeth and facilitate controlled movement toward the lesser segment. A digital arch form was defined, and progressive movement of 0.5 mm per aligner was programmed to guide cleft closure (Figure 2).

All impressions, scans, and morphometric measurements were performed by the same examiner to ensure consistency. To evaluate intra-examiner reliability, a random 20% subset of casts was re-landmarked after a two-week interval. Agreement was assessed using Bland–Altman analysis (bias and 95% limits of agreement), the repeatability coefficient (RC), and the technical error of measurement (TEM and %TEM). Reliability was considered acceptable when bias was close to zero, limits of agreement were narrow relative to treatment effects, and %TEM was <5% (excellent) or <10% (acceptable).

### 2.3. Model Printing and Aligner Fabrication

Based on the digital plan, physical models were printed in opaque resin (MED 620; Stratasys Ltd. Eden Praire, MN, USA) at 16 µm resolution. Thermoformed aligners were fabricated using a three-layer elastic-plastic material (Ultimate; Compass 3D–GmbH, Benshein, Germany), composed of PET-G outer layers and a thermoplastic core (0.7 mm total thickness). Only the initial sequence of aligners (Phase I) was fabricated and delivered at this stage [18] (Figure 2).

### 2.4. Appliance Delivery and At-Home Protocol

**Figure 3:**
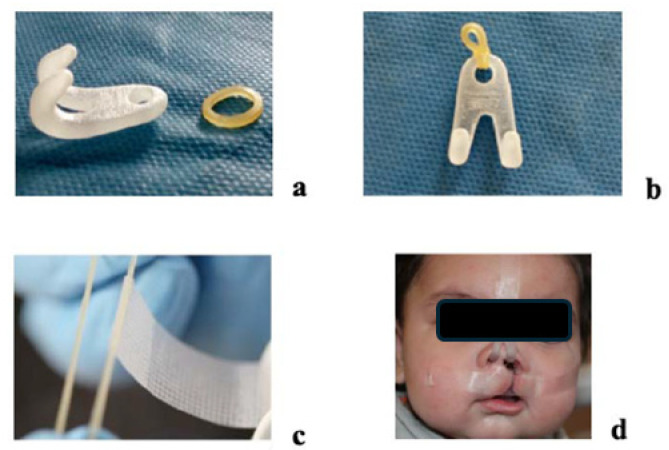
Nasal protractor. All quantitative measurements were obtained from digital 3D models and are reported in the Results; therefore, no scale bars are shown in the photographs. (**a**) Acrylic nasal retractor with a 3/16-inch rubber band. (**b**) Rubber band attached to the nasal retractor. (**c**) Tape wrapped around the rubber band. (**d**) Nasal retractor delivered to the patient with additional lip taping.

At the second visit, the first aligner was fitted and adjusted using rotary instruments and polishing tools. Caregivers were instructed to replace aligners weekly and to use denture adhesive (Corega Gel; GSK, Brazil Ltd.a., Rio de Janeiro, Brazil) if retention was insufficient. In addition to the intraoral appliance, daily lip taping (Nexcare 12 mm × 4.5 mm; 3M) was prescribed to assist with anterior segment approximation (Figure 3). Parents were advised on gentle tape removal using water or mineral oil to prevent skin irritation [18].

A nasal protractor was introduced concurrently, consisting of forehead-anchored support, elastic retention, and intranasal hooks. Caregivers monitored the tension and placement to ensure consistent nasal molding throughout the course of treatment (Figure 3).

### 2.5. Midpoint Evaluation and Phase II

Upon completion of Phase I, a second impression was taken to account for maxillofacial growth. A new digital plan (Phase II) was created, and a second set of aligners was fabricated and delivered at the fourth visit, using the same clinical and fabrication procedures.

### 2.6. Final Visit and Post-Treatment Records

At the final appointment, the patient was examined for completion of the orthopedic objectives. The last aligner was used as a passive retainer, to be worn along with the lip tape and nasal protractor until the time of lip surgery. The parents were instructed to use the final clear aligner as a retainer (Figure 2f) in conjunction with the lip tape and the nasal retractor until the patient underwent surgery. Lastly, a post-NAM (T2) impression was taken and scanned into OrthoAnalzer, where it was compared to the pre-NAM (T1) impression [18].

**Figure 4:**
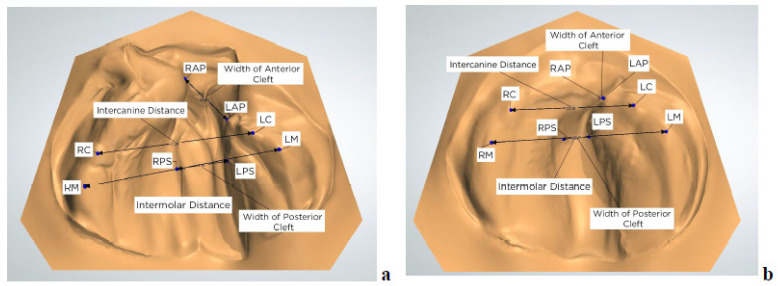
Anatomical reference points and linear measurements. (**a**) Measurements before clear aligner NAM treatment. (**b**) Measurements after clear aligner NAM treatment. RAP/LAP (right/left alveolar point)—corresponds to the most anterior and medial point on the patient’s right/left alveolar ridge segment; RC/LC (Right/left canine)—corresponds to the most superior point on the alveolar ridge in the region of the estimated right/left deciduous canine; RM/LM (Right/left molar)—corresponds to the most superior point on the alveolar ridge in the region of the estimated right/left deciduous molar; RPS/LPS (Right/left palatal shelf)—distance from RM/LM to the palatal cleft, along the line that connects points RM and LM; Anterior Cleft Width—distance between points RAP and LAP; Intercanine Distance—distance between points RC and LC; Posterior Cleft Width—measured from the most medial point of the right segment to the most medial point of the left segment, along the line that connects RM and LM; Intermolar Distance—distance between points RM and LM.

For each of the 25 subjects, four measurements were obtained: anterior cleft width, posterior cleft width, intercanine width, and intermolar width (Figure 4). All measurements were taken in OrthoAnalyzer (3Shape) and recorded in an Excel sheet. To obtain these measurements, specific anatomic points were defined and used [18].

### 2.7. Measurements and Statistical Analysis

Anatomical measurements were performed using OrthoAnalyzer software (3Shape) based on standardized reference points on the 3D digital models (Figure 4). Anterior cleft width was defined as the distance between the right and left alveolar points (RAP and LAP), located at the most anterior and medial aspects of the greater and lesser alveolar segments. Posterior cleft width was measured between the most medial points of the posterior maxillary segments, along the line connecting the right and left molar points (RM and LM).

Intercanine width was measured between the right and left canine points (RC and LC), corresponding to the highest points on the alveolar ridge in the region of the estimated primary canines. Intermolar width was defined as the transverse distance between RM and LM, representing the crests of the alveolar ridge in the region of the future primary first molars.

Due to anatomical variability in neonates with cleft lip and palate, particularly near the cleft margins, some of these reference points—especially RAP, LAP, RM, and LM—were considered semi-landmarks, placed according to consistent visual and anatomical criteria to ensure reproducibility across cases and time points (Figure 4).

All measurements were collected at baseline (T1) and after the completion of treatment (T2) [18]. Statistical analysis was conducted using SPSS software (Version 25.0; IBM Corp., Armonk, NY, USA). Paired *t*-tests were used to evaluate changes between T1 and T2. A *p*-value of <0.05 was considered statistically significant. Given the exploratory nature of this retrospective series and the limited sample size, no formal correction for multiple testing (e.g., the Bonferroni method) was applied. This increases the potential risk of Type I error, and findings should be interpreted with caution.

## 3. Results

Treatment was completed in an average of 5.0 ± 0.8 visits over a period of 19.8 ± 1.7 weeks. All patients resided in rural areas and traveled an average distance of 103 ± 24 km to the treatment center. No adverse effects were observed during the study period; only occasional, mild redness at the taping site was noted, without any associated discomfort or parental concerns.

### 3.1. Quantitative Outcomes

The most significant anatomical change occurred in the anterior cleft width, which decreased from 10.02 mm at baseline (T1) to 4.64 mm at the end of treatment (T2). This corresponded to a mean reduction of 5.38 mm (95% CI: –7.58 to –3.18, t = 5.06, *p* < 0.001). The largest reduction observed in a single case was from 10.25 mm to 0.37 mm, representing a 96% closure of the cleft. (Table 1).

The posterior cleft width also decreased significantly, from 11.60 mm at T1 to 8.20 mm at T2, yielding a mean reduction of 3.39 mm (95% CI: –4.79 to –1.99, t = 5.01, *p* < 0.001).

The intermolar distance increased significantly, from 35.34 mm at T1 to 36.57 mm at T2, with a mean increase of 1.23 mm (95% CI: 0.44 to 2.02, t = –2.21, *p* = 0.036). This suggests that transverse arch development was preserved and likely enhanced during therapy. (Table 1)

In contrast, the intercanine width showed only a minor change, increasing from 27.01 mm to 27.50 mm, with a mean difference of 0.49 mm (95% CI: 0.05 to 2.04, t = –0.66, *p* = 0.515), indicating that anterior arch form integrity was maintained throughout treatment. The confidence interval for intercanine distance (0.05–2.04 mm) suggested a possible small increase, but this difference was not statistically significant, reflecting sample variability and the limited magnitude of change. (Table 1).

The calculations were verified, and no error was identified. To contextualize these results, effect sizes (Cohen’s d) and per-patient change ranges are provided in Supplementary Table S1, illustrating the magnitude of treatment effects and variability across patients. Large effect sizes were observed for anterior and posterior cleft width reduction [Cohen’s dz > 1.0]. Intra-examiner reliability testing demonstrated negligible bias, narrow 95% limits of agreement, and low repeatability coefficients across all variables. TEM and %TEM values were small, depending on the measurement, confirming that measurement error was minimal relative to the observed treatment changes. (Supplementary Table S2).

### 3.2. Qualitative Observations

**Figure 5:**
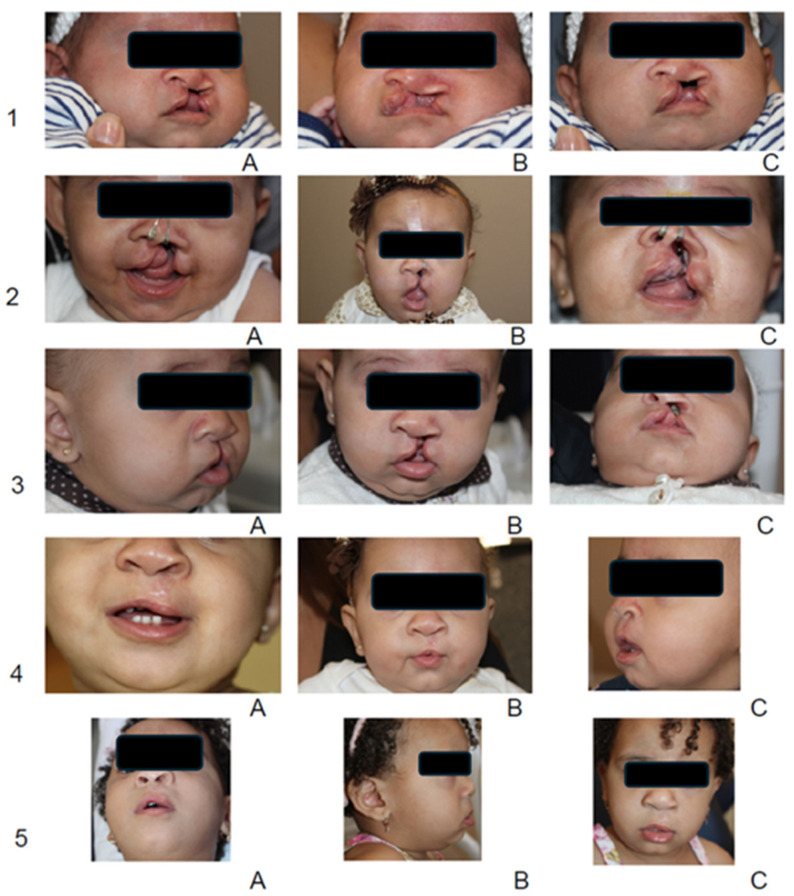
Analysis of pre- and post-NAM treatment and post-surgery results. Although minor variations in angle and lighting occurred during routine clinical documentation, these images were selected to illustrate treatment progress. Quantitative outcomes were derived from 3D models rather than from clinical photographs. Sequential extraoral photographs illustrate five key time points: (1) A–C, pretreatment; (2) A–C, midway through NAM clear aligner therapy; (3) A–C, at completion of therapy; (4) A–C, one-month post-surgery; and (5) A–C, five months post-surgery. The images demonstrate progressive approximation of the lip segments, improved nasal symmetry, increased columellar length, and satisfactory esthetic healing following primary lip repair.

Clinicians noted and reported the observations demonstrating consistent improvements in nasal symmetry, columellar length, and reduced visible scarring at the time of primary lip repair. Surgeons reported that the approximation of lip segments, effective in reducing cleft width over the 20-week pre-surgical period, enabled more conservative and efficient procedures, facilitating more favorable nasal tip positioning (Figure 5). However, it is essential to note that objective nasal measurements were not collected, which limits the quantitative assessment of nasal symmetry.

## 4. Discussion

This clinical study evaluated the feasibility and outcomes of a digitally guided nasoalveolar molding (NAM) technique using clear aligners in infants with unilateral complete cleft lip and palate from rural, low-income regions. The protocol, designed to address barriers inherent in traditional NAM—such as frequent in-office visits and limited access to trained providers—yielded both statistically and clinically meaningful improvements. Only the first half of the aligners were printed initially in order to allow for a scheduled follow-up visit at mid-treatment. This approach ensured that infant growth and treatment progress could be evaluated before fabricating the remaining appliances, providing better clinical control and adaptability.

Quantitatively, the most significant anatomical change occurred in the anterior cleft region, where a mean reduction of 5.38 mm was observed (95% CI: –7.58 to –3.18, *p* < 0.001). This finding supports the efficacy of programmed medial movement and rotation of the digitally segmented greater maxillary segment. Posterior cleft width also decreased significantly, with a mean reduction of 3.39 mm (95% CI: –4.79 to –1.99, *p* < 0.001). This change may be attributable to the palatal obturation effect of the thermoformed aligner, which likely helped limit lateral pressure from the tongue and supported medial segment growth [7].

The intermolar width increased by 1.23 mm over the course of treatment (95% CI: 0.44 to 2.02, *p* = 0.036), indicating that transverse arch development was maintained and possibly enhanced. This finding supports the protocol’s capacity to prevent posterior arch constriction, which is a potential concern in orthopedic cleft therapy. Because multiple paired *t*-tests were conducted without correction for multiplicity, some significant findings, particularly intermolar width changes, may be vulnerable to Type I error. In contrast, the intercanine width remained stable, with a minimal mean increase of 0.49 mm (95% CI: 0.05 to 2.04, *p* = 0.515), suggesting that anterior arch form integrity was preserved during cleft segment approximation. This is a noteworthy outcome, as the collapse of the anterior arch has been reported in some traditional presurgical orthopedic protocols [9].

In the absence of concurrent control, we compared our effect sizes with those of historical cohorts. Prior NAM studies report mean preoperative reductions in anterior/alveolar cleft width of approximately 3–8 mm, often exceeding changes seen with passive plates or relative to birth status. Our anterior (–5.38 mm) and posterior (–3.39 mm) cleft width decreases fall within this range, while small transverse gains (intermolar +1.23 mm; intercanine +0.49 mm) align with published patterns of modest arch widening during therapy. Together, these data suggest the magnitude of change in this study is comparable to historical NAM outcomes [19]. Recent comparative and systematic reviews indicate that NAM typically achieves greater narrowing of the anterior segment than passive appliances, and large randomized trials are underway to more definitively quantify the benefit versus no NAM [20,21].

While our analysis focused primarily on anatomical outcomes, previous NAM studies have also highlighted potential functional benefits such as improved feeding efficiency and early weight gain in infants with cleft lip and palate. These outcomes were not systematically assessed in our cohort, and our findings should therefore be interpreted in light of this limitation. In addition, caregivers in our series frequently expressed a sense of reassurance and empowerment when participating in the daily appliance management. These impressions were anecdotal rather than measured systematically, but prior studies have documented psychosocial benefits of presurgical molding, including reduced parental stress and improved bonding with the infant [22,23,24,25]. Future prospective investigations should incorporate standardized measures of feeding and validated caregiver-reported outcome tools to provide a more comprehensive understanding of the broader impact of digital presurgical molding. Subjectively, surgeons observed improved nasal symmetry, increased columellar length, and reduced intraoperative tissue tension, which facilitated more conservative and efficient primary lip repairs. (These clinical observations are in agreement with previous findings that NAM improves nasal shape and reduces soft tissue distortion, ultimately enhancing surgical outcomes [2,3,4,5,6].

This protocol’s use of a separate nasal protractor, rather than an integrated stent, introduced two key advantages: (1) nasal molding could be initiated early—before maxillary segments approached each other within 5 mm—thus leveraging the high malleability of postnatal nasal cartilage [6]; and (2) nasal shaping could be continued independently throughout the preoperative period, offering flexibility and sustained correction.

Beyond anatomical and surgical outcomes, the protocol demonstrated practical benefits for families in remote areas. Patients lived over 100 km from the clinic but completed treatment in approximately five visits over 20 weeks. In contrast, traditional NAM often requires 13–14 weekly visits over a similar time span [17]. This digitally guided approach reduces travel burden and dependency on specialized personnel, making it especially relevant for implementation in low- and middle-income countries [13,14,15,16].

Additionally, the model promoted family engagement. Parents actively participated in appliance changes and nasal taping and were able to visualize treatment progression through the digital planning interface. This participatory model enhanced communication and may have contributed to the strong adherence observed. Caregivers also reported a sense of empowerment—an outcome that aligns with literature supporting the psychosocial benefits of parent-led NAM interventions [10,11,12,15,18].

This digital NAM clear aligner protocol has potential for use in regions where conventional NAM is challenging due to distance, access to trained providers, or costs. Recent reports from Egypt and other LMIC contexts have demonstrated that 3D-printed and vacuum-formed NAM approaches can be implemented effectively in resource-limited environments, supporting the feasibility of broader international application [26,27]. However, conclusions regarding scalability must remain cautious until cost-effectiveness and implementation studies are performed.

### Limitations

This study is retrospective, and as such is subject to important limitations, including potential selection bias, recall bias, and the lack of prospective standardization. We therefore contextualized our findings against published historical cohorts; however, such comparisons are susceptible to differences in protocols and case mix. The use of semi-landmarks in digital models introduces the potential for measurement variability; however, all measurements were performed by a single examiner, which ensured consistency but did not allow evaluation of inter-examiner reproducibility. Nevertheless, intra-examiner repeatability analyses indicated that measurement error was small compared with the magnitude of the treatment changes. The absence of a control group limits direct comparison with traditional NAM, and the modest sample size, while in line with similar clinical studies, restricts generalizability. As a retrospective series without an a priori power calculation, the study is limited by the available sample size; post hoc power is reported solely for transparency and should be interpreted with caution.

Because several paired *t*-tests were performed without correction for multiple testing, there is an increased risk of Type I error. This reflects the exploratory design and limited sample size; therefore, the results should be interpreted accordingly. Nasal symmetry outcomes were assessed qualitatively, rather than measured using objective tools, which limits quantitative assessment of nasal symmetry and reduces comparability with other studies [4,6,11]. In addition, no implementation or cost-effectiveness data were collected; therefore, any statements regarding scalability should be considered speculative. This approach was feasible and effective in reducing cleft width during the 20-week pre-surgical period. While qualitative surgeon feedback suggested improved nasal form, this was not objectively measured and represented an area for future investigation. Broader claims regarding scalability and accessibility remain speculative, as formal implementation strategies and cost-effectiveness analyses were not undertaken in this study. Future research should aim to include larger, multicenter cohorts, control arms, and long-term follow-up to assess post-surgical outcomes and craniofacial growth trajectories.

## 5. Conclusions

This clinical study demonstrates that a digitally guided nasoalveolar molding (NAM) protocol using clear aligners is a feasible, effective, and accessible alternative to traditional NAM, particularly in rural and low-resource settings. The protocol produced significant reductions in anterior cleft width (–5.38 mm, *p* < 0.001) and posterior cleft width (–3.39 mm, *p* < 0.001), without compromising anterior arch form. The increase in intermolar width (+1.23 mm, *p* = 0.036) further supports that transverse maxillary development was maintained during therapy.

The stable intercanine width and favorable qualitative outcomes, including improved nasal symmetry and columellar length, suggest that this protocol not only improves anatomic alignment but also facilitates more conservative and efficient surgical repairs. Additionally, the low number of clinical visits and the active role of caregivers make this approach particularly suitable for underserved regions with limited access to cleft care.

As 3D imaging, digital planning, and additive manufacturing become more widely available, this model may serve as a scalable framework for expanding early cleft care globally. In summary, the digital NAM clear aligner protocol appears feasible and promising, with encouraging results in reducing cleft width. While this approach has potential for broader implementation, especially in regions where conventional NAM is challenging, conclusions about global scalability should be made with caution. Data on cost-effectiveness, workforce training, and implementation in resource-limited settings are still lacking, and future studies are needed to address these critical aspects before widespread adoption can be recommended. Further prospective studies are needed to validate long-term outcomes and support broader adoption of digitally guided NAM protocols in multidisciplinary cleft care teams.

## Figures and Tables

**Table 1:** Statistical results of anatomical measurements Pre- and Post- NAM clear aligner treatment (T1 and T2).

Measurement	Average at T1	Average at T2	Difference (T2–T1)	95% CI	t-Value	*p*-Value
Anterior Cleft Width (mm)	10.02	4.64	−5.38	(−7.58, −3.18)	5.06	<0.001 *
Intercanine Distance (mm)	27.01	27.5	0.49	(0.05, 2.04)	−0.66	0.515
Posterior Cleft Width (mm)	11.6	8.2	−3.39	(−4.79, −1.99)	5.01	<0.001 *
Intermolar Distance (mm)	35.34	36.57	1.23	(0.44, 2.02)	−2.21	0.036 *

Differences are reported as T2–T1, while t-values reflect the software’s default test direction (T1–T2); therefore, signs may differ, though magnitudes and *p*-values are identical. * Statistically significant (*p* < 0.05).

## Data Availability

Data is unavailable due to privacy or ethical restrictions.

## Supplementary Materials

The following supporting information can be downloaded at https://www.mdpi.com/article/10.3390/children12091231/s1, Figure S1: Patient Flow Diagram; Table S1: STROBE Checklist for Observational Studies; Table S2. Effects and Per-patient Change Ranges.
