# Supplementary material for: Expanding Access to Presurgical Cleft Care: Digital Nasoalveolar Molding with Clear Aligners in a Rural Low-Income Population
Source: Children (Basel). 2025 Sep 15;12(9):1231. doi: 10.3390/children12091231 (PMC12468677; doi:10.3390/children12091231)
Supplement: Supplementary file 1 [file children-12-01231-s001.zip › children-3829864-supplementary-english.pdf]

Supplementary Table S1. STROBE Checklist for Observational Studies

Study type: Retrospective observational study of presurgical molding in infants with cleft lip and palate.

Item	Recommendation	Addressed in Manuscript (Section)
Title and Abstract	Indicate study design in title/abstract	Title and Abstract (design noted as 'retrospective observational')
1. Background/Rationale	Explain scientific background and rationale	Introduction
2. Objectives	State specific objectives/hypotheses	Introduction (last paragraph)
3. Study Design	Present key elements of study design early	Methods → Study Design
4. Setting	Describe setting, locations, and dates	Methods → Setting and Study Period
5. Participants	Eligibility criteria, sources, number of participants	Methods → Participants
6. Variables	Define all outcomes, exposures, predictors	Methods → Outcomes and Measurements
7. Data Sources/Measurements	Explain how data were collected/measured; comparability of groups	Methods → Cast analysis and measurements
8. Bias	Efforts to address potential bias	Methods → Reliability testing; Limitations
9. Study Size	Explain how study size was determined	Methods → Sample size (all eligible patients included)
10. Quantitative Variables	Explain handling of quantitative variables	Methods → Statistical Analysis
11. Statistical Methods	Describe statistical methods; handling of confounding, missing data, subgroup analyses	Methods → Statistical Analysis
12. Participants (Results)	Report numbers at each stage of study; reasons for exclusions	Results → Participant characteristics
13. Descriptive Data	Characteristics of study population	Results → Table of demographics (if applicable)
14. Outcome Data	Report numbers of outcome events or summary measures	Results → Table X
15. Main Results	Give unadjusted estimates, confidence intervals, and p-values	Results → Table X, Results text
16. Other Analyses	Subgroup, sensitivity analyses if applicable	N/A (not performed)
17. Key Results	Summarize main results with reference to study objectives	Discussion → first paragraph
18. Limitations	Discuss study limitations, bias, imprecision	Discussion → Limitations
19. Interpretation	Provide cautious interpretation of results	Discussion → Interpretation
20. Generalizability	Discuss external validity	Discussion → Limitations/Conclusions
21. Funding	Source of funding and role of funders	Acknowledgments / Funding statement

Supplementary Table S2. Effects and Per-patient Change Ranges

Measurement	Mean Difference (T2–T1, mm)	95% CI of Difference	Effect Size (Cohen's d_z)	Per-patient Change Range (mm)	Sample Size (n)
Anterior Cleft Width (mm)	-5.09	(-7.58, -3.18)	-1.03	(-14.0, 6.32)	25
Intercanine Distance (mm)	0.28	(0.05, 2.04)	0.15	(-4.34, 4.01)	25
Posterior Cleft Width (mm)	-3.49	(-4.79, -1.99)	-1.02	(-8.49, 4.39)	25
Intermolar Distance (mm)	0.91	(0.44, 2.02)	0.5	(-2.03, 6.07)	25
