# Supplementary figures and images for: Expanding Access to Presurgical Cleft Care: Digital Nasoalveolar Molding with Clear Aligners in a Rural Low-Income Population
Source: Children (Basel). 2025 Sep 15;12(9):1231. doi: 10.3390/children12091231 (PMC12468677; doi:10.3390/children12091231)

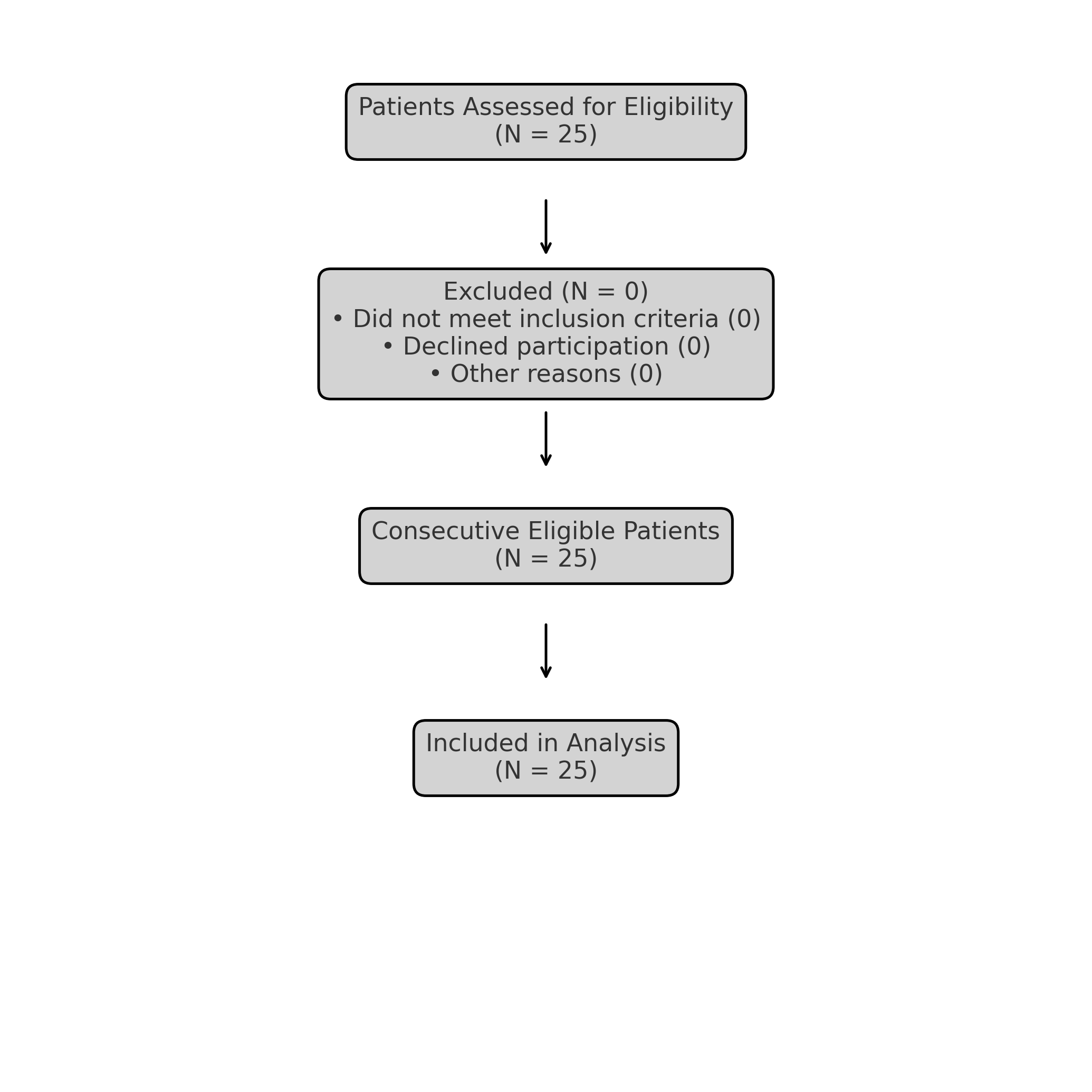

Supplement: Supplementary file 1 [file children-12-01231-s001.zip › children-3829864-supplementary.png]
